# Boosting the Thermoelectric Performance of Pseudo‐Layered Sb_2_Te_3_(GeTe)*_n_* via Vacancy Engineering

**DOI:** 10.1002/advs.201801514

**Published:** 2018-10-12

**Authors:** Xiao Xu, Lin Xie, Qing Lou, Di Wu, Jiaqing He

**Affiliations:** ^1^ Shenzhen Key Laboratory of Thermoelectric Materials Department of Physics Southern University of Science and Technology Shenzhen 518055 China; ^2^ School of Materials Science and Engineering Shaanxi Normal University Xi'an 710119 China

**Keywords:** in situ TEM, Sb_2_Te_3_(GeTe)*_n_*, thermoelectric properties, vacancy engineering

## Abstract

An ultrahigh figure of merit ZT value ≈2.4 at 773 K for p‐type pseudo‐layered Sb_2_Te_3_(GeTe)_17_ along the parallel direction is reported by synergistically optimizing its electrical and thermal properties via vacancy engineering. The microstructural origin of thermoelectric property enhancement is studied by spherical aberration corrected transmission electron microscopy and its in situ mode. The results reveal that upon annealing, Ge vacancy gaps in quenched samples tend to migrate and recombine into long‐range gaps in order to minimize the elastic and electrostatic energies. The recombination of Ge gaps would lead to an overall reduction of carrier concentration and electrical thermal conductivity. The detailed study of Ge vacancies migration via heat treatment and its effects on thermoelectric performance in pseudo‐layered Sb_2_Te_3_(GeTe)_17_ materials can provide enlightening clues for future research in a number of thermoelectric materials of similar structures.

Thermoelectric materials have drawn the attentions of worldwide researchers for their unique functionality by converting the waste heat into electricity directly.^1^, ^2^, ^3^ The performance of a thermoelectric material is usually characterized by the dimensionless figure of merit, ZT *= α* ^2^ *σT*/κ, where *α, σ, T*, and κ are the Seebeck coefficient, electrical conductivity, absolute temperature, and thermal conductivity, respectively. Thus, materials with a large power factor (*α^2^σ*)^4^, ^5^ which minimizes the Joule heating, and a low total thermal conductivity (κ)^6^, ^7^ which maintains the temperature gradient, are expected in order to maximize the ZT value. As demonstrated in pioneering work, power factor can be improved by means of band convergence,^8^ carrier energy filtering,^9^ and resonant energy,^10^ while low thermal conductivity can be realized through the introduction of point defects,^11^, ^12^ second phases,^13^ boundaries, and precipitates.^14^

The IV–VI group chalcogenides have been deeply investigated as promising intermediate temperature thermoelectric materials. However, the narrow band gap semiconductor GeTe is rarely considered to be a suitable material because of its intrinsically high carrier concentration (≈10^21^ cm^−3^).^15^, ^24^ Thus, methods of reducing the carrier concentration^16^, ^17^, ^18^ in the GeTe based sample are investigated persistently. Lee et al.^19^ have investigated the properties of Mn‐doped GeTe and showed that the increment of electrical properties was due to the reduction of carrier concentration. Li et al.^20^ demonstrated that the carrier concentration could be reduced with Pb doping. Besides, the lattice thermal conductivity of GeTe can be significantly reduced by alloying with AgBiTe_2_,^21^ In_2_Te_3_,^22^ and AgSbTe_2_.^23^ In particular, Wu et al.^24^ obtained a peak ZT value of ≈1.9 at 773 K in 3 mol% Bi_2_Te_3_ alloyed Ge_0.87_Pb_0.03_Te samples and attributed the high electrical properties to the enhanced band degeneracy of the light and heavy valence bands with Bi_2_Te_3_ alloying.

Recently, the pseudo‐layered GeTe‐rich Sb_2_Te_3_(GeTe)*_n_* (GST), which has a similar phase transition behavior as GeTe, i.e., from the cubic phase (high temperature) to the rhombohedral phase (room temperature), is found to have promising thermoelectric performance.^25^, ^26^ With a unique Te–Te gaps in the unit cell, Sb_2_Te_3_(GeTe)*_n_* sample usually exhibits a lower lattice thermal conductivity in comparison to GeTe, which makes this system a competitive one in thermoelectric field.^27^, ^28^, ^29^, ^30^ The thermoelectric properties of several types of GeTe‐rich GST materials have been studied, for example, GeSb_6_Te_10_,^27^ Ge_7_Sb_2_Te_10_,^28^ Ge_12_Sb_2_Te_15_,^29^ and Ge_19_Sb_2_Te_22_.^30^ Fahrnbauer et al.^31^ found that the introduction of CoGe_2_ precipitates into the intrinsic nanostructures could decrease the total thermal conductivities, which results in a maximum ZT value up to 1.8 for (CoGe_2_)_0.22_Sb_2_Te_3_(GeTe)_19_ samples. It is also reported by Williams and Morelli^32^ that the Ge_17_Sb_2_Te_20_ samples exhibit high thermoelectric performance of ≈2.0, but the mechanism is still unclear.

In this work, an ultrahigh figure of merit of 2.4 at 773 K along the parallel direction is obtained by successfully tuning Ge vacancies in pseudo‐layered Sb_2_Te_3_(GeTe)_17_ via suitable thermal treatments. A scenario involving dynamical migration, recombination and structure reconstruction of Ge vacancies upon annealing is clearly demonstrated by in situ transmission electron microscopy (TEM) and atomic‐resolution scanning TEM (STEM). We attribute the high ZT value to 1) the optimized carrier concentration due to the appropriate annealing process, 2) the relatively low lattice thermal conductivity caused by the intrinsic and reformed gaps, and 3) the high symmetry structure at high temperature region. We believe the investigation of vacancy engineering in this work can shed light on development of GST‐based thermoelectric materials.

We synthesized a series of Sb_2_Te_3_(GeTe)*_n_* samples with Spark Plasma Sintering process. The X‐ray diffraction pattern of Sb_2_Te_3_(GeTe)*_n_* bulk samples with different GeTe rich compounds (*n* = 7, 12, 14,17, and 19) is shown in Figure S1 in the Supporting Information, implying that all the samples show a totally different phase with GeTe. We chose the composition with *n* = 17 for further investigation because this sample exhibits a better electrical property and an appropriate thermal property compared with the other compounds, as shown in Figure S2 in the Supporting Information. Considering the low symmetry and anisotropy of the low temperature phase, we conducted electrical and thermal transport characterizations in samples with *n* = 17 along parallel and perpendicular to the press direction, individually, as shown in Figure S13 in the Supporting Information. Results implied only slight difference can be defected along the two directions, i.e., ZT in the parallel direction is little superior than that in the perpendicular direction. Thereafter, we focused on the parallel direction only in this work. A high ZT value of ≈2.0 is reached at 723 K, which is consistent with the literature about Sb_2_Te_3_(GeTe)_17._ 32

Sb_2_Te_3_(GeTe)_17_ samples are fabricated and annealed at 873 K with 0, 2, 4, and 7 days, respectively. The X‐ray diffraction (XRD) patterns of these samples are illustrated in Figure S3 in the Supporting Information, and the differential scanning calorimetry (DSC) curves have been given in Figure S4 in the Supporting Information. Both details are discussed in the Supporting Information to indicate that annealing method is of great benefit to reduce the phase transition temperature *T*_c_, which is proved an effective way to enhance the electrical properties in phase change materials GeTe by Hong et al.^33^ In order to understand how the annealing method affects the electrical properties, a series of measurements are performed. As presented in **Figure** **1**a, the electrical conductivity of Sb_2_Te_3_(GeTe)_17_ sample before annealing (red line) is much higher than the other samples in the whole temperature range, and it is clear that there is a phase change near 610 K, which is consistent with the DSC results. As the annealing time increases, the feature of phase transition appears earlier than anticipated and the electrical conductivities decreases abruptly. For the 7 days' annealing samples (blue lines), the room temperature electrical conductivity is near 700 S cm^−1^, just half of the sample before annealing. As shown in Figure 1b, the Seebeck coefficient reaches a nearly largest value of 250 µV K^−1^ after 7 days' annealing at high temperature, compared with a value less than 200 µV K^−1^ before annealing. As a result, the 7 days' annealed sample obtains a better power factor, especially in the high temperature phase area. A high value plateau of power factor from 623 to 773 K can be reached as shown in Figure 1c.

**Figure 1:**
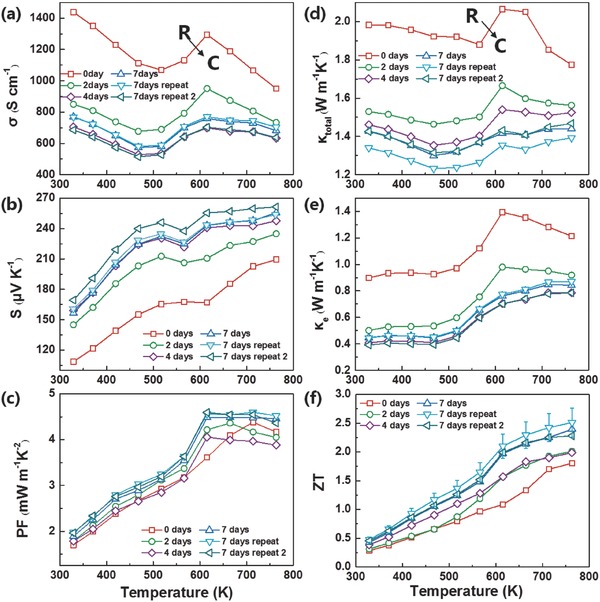
The temperature dependence of a) the electrical conductivity, b) the Seebeck coefficient, c) the power factor, d) the total thermal conductivity, and e) the electrical thermal conductivity and f) the ZT value.

Figure 1d shows the temperature‐dependent total thermal conductivity (κ_total_) of before and after annealing. Before annealing, the room temperature thermal conductivity value is near 2.0 W m^−1^ K^−1^. After annealing, it decreases by about 30%, with an average value of three samples for ≈1.4 W m^−1^ K^−1^ after 7 days' annealing. What's more, annealing seems to have minor effects on thermal conductivities in the cubic phase area and the thermal conductivity of the sample annealed for 7 days remains constant at the temperature range from 623 to773 K. The diffusivities of all the samples (Figure S5a, Supporting Information) have a similar performance as the total thermal conductivities. The electronic part of the total thermal conductivity is calculated according to the Wiedemann–Franz law: κ_e_ = *LσT*, where *L*, the Lorenz number, could be calculated by combining the experimental Seebeck coefficient of each sample in a single parabolic band model,^34^ shown in the Figure S5b in the Supporting Information. The calculated κ_e_ is displayed in Figure 1e. As is expected from the large reduction of electrical conductivity, the electronic thermal conductivity reduces substantially. As a result, this part of thermal properties got a prominent optimization. The lattice thermal conductivities (κ_lattice_) are calculated by the equation: κ_lattice_ = κ_total_ − κ_e_ and shown in Figure S5c in the Supporting Information. The κ_lattice_ for samples with 7 days' annealing is reduced by 10%–20% compared with the nonannealed sample at the room temperature. However, almost all annealed samples have a larger lattice thermal conductivity than that of the nonannealed one in the high‐temperature region (623–773 K). This phenomenon seems contradictory in the materials but consistent with the TEM results, and a reasonable explanation will be given in a later part. The outstanding performance of the Sb_2_Te_3_(GeTe)_17_ samples is presented in Figure 1f. As the annealing time increases, the ZT value becomes higher, and the maximum ZT value of the sample which is annealed for 7 days reaches ≈2.4 at 773 K with good reproducibility. The stability test has been done in the 7 days' annealing sample. We heat it and cool down with another three times and the thermoelectric performance is shown in Figure S6 in the Supporting Information. Neither the electrical properties nor the thermal properties deteriorate during the heating and cooling processes which suggests an excellent stability of our specimens.

The carrier concentration‐dependent Seebeck coefficient of our Sb_2_Te_3_(GeTe)_17_ samples is fitted to the Pisarenko line calculated with a single Kane band model, **Figure** **2**a. The distinct fitting effective masses between Sb_2_Te_3_(GeTe)_17_ and reported GeTe samples imply that they shall own very different valence band structures, which shall be intrinsic to the pseudo‐layered Sb_2_Te_3_(GeTe)*_n_* rather than a slight modification on GeTe. Notably, Sb_2_Te_3_(GeTe)_17_ samples exhibit much larger effective mass, m*, than that of GeTe, favorable for higher *S* values. The exact valance band structure of Sb_2_Te_3_(GeTe)_17_ needs further investigations with density functional theory calculations or angle‐resolved photoemission spectroscopy characterizations, which exceed the scope of this work. Figure 2b presents the annealing time‐dependent carrier concentration and mobility. Figure 2c exhibits the dependence of carrier concentration and mobility on temperature. The carrier concentration decreases as the annealing time increases, suggesting that this pseudo‐layered Sb_2_Te_3_(GeTe)_17_ system is sensitive to the heat treatment process. The carrier concentration of Sb_2_Te_3_(GeTe)_17_ sample before annealing meets a sharply increasing whereas after annealing the change goes to a deceleration. There is no obvious growth as the temperature increases after annealing because the sample tends to be a comparatively lower energy state. As a result, the hole density of the sample before annealing is up to ≈10^21^ cm^−3^, while after 7 days' annealing it is only a quarter. For the temperature‐dependent carrier mobility shown in Figure 2d, the values of the four samples fluctuate around 15 cm^2^ V^2^ s^−1^. According to the reported literatures,^16^ the carrier concentration in GeTe system originates largely from the intrinsic Ge vacancies. Thus, the decreasing carrier concentration as the annealing time indicates the reduction of Ge vacancies.

**Figure 2:**
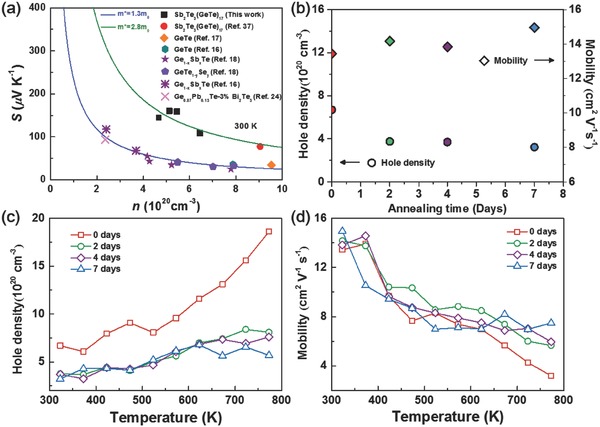
a) The carrier concentration dependence of Seebeck coefficient of Sb_2_Te_3_(GeTe)_17_ and GeTe, b) the annealing time dependence of hole density and mobility and the temperature dependence of c) the hole density and d) the mobility.

To validate the deduction above, we conducted microstructural studies on Sb_2_Te_3_(GeTe)_17_ samples before and after annealing via C_S_‐corrected STEM high‐angle annular dark field (HAADF) imaging. As clearly shown in **Figure** **3**a, there is a high density of short Ge planar vacancies in the Sb_2_Te_3_(GeTe)_17_ matrix before annealing (marked by yellow arrows). Low magnified TEM images from another direction in the same grain and the images of two different grains were also provided in the Figure S7 in the Supporting Information. A good homogeneity of these planar defects was revealed. The atomic resolution experimental image of the defect and the corresponding simulated HAADF image are shown in the inset in Figure 3a. A slight change of Ge atoms' contrast indicates that Ge vacancies tend to segregated in the equivalent {0001} plane. STEM‐HAADF image simulation is also performed and a structure with 30% Ge vacancies in one of (0001) plane is simulated (inset, Figure 3a). The calculated STEM image is in good agreement with the experimental result, which confirms the partial segregation of Ge vacancies in the quenched samples. In comparison, Figure 3b shows the HAADF image for the Sb_2_Te_3_(GeTe)_17_ material with 7 days' annealing. Different from the quenched sample, the size of Ge vacancies segregated in {0001} plane becomes much larger and their length can be up to around several hundred nanometers. Moreover, the image contrast corresponding to the Ge vacancy plane becomes negligible and a substantial structural reconstruction by means of a relative shift along 1/3[120] direction between adjacent Te atomic planes could be readily observed (shown in the inset, Figure 3b), suggesting the formation of a complete segregation of Ge vacancies which is in analog to van der Waals gaps in 2D materials. STEM HAADF image simulation of the atomic structure with a single layer of Ge vacancies and shift between adjacent Te planes are also carried out and the simulated image (shown in inset, Figure 3b) matches the experimental results very well.

**Figure 3:**
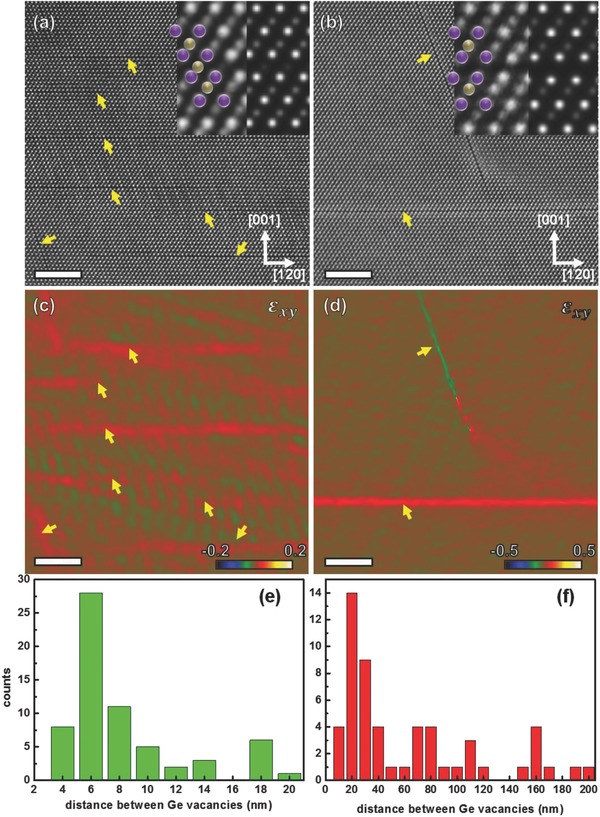
The HRTEM images of the Sb_2_Te_3_(GeTe)_17_ samples a) without annealing and b) with 7 days' annealing, left inset shows magnified part of the structure around the Ge vacancies and right one shows the simulated result in the same area, the scale bar is 10 nm. The GPA strain analysis images of the same region of the HRTEM images in the c) nonannealed sample and d) 7 days' annealing sample. Panels (e) and (f) are the histograms of distances between Ge vacancies in the two samples, respectively.

Figure 3c,d shows the corresponding geometric phase analysis (GPA) strain analysis results of Figure 3a,b, respectively (a complete strain analysis in all directions is given in Figure S8 in the Supporting Information). Obviously, the strain field of the defects for the samples annealed for 7 days becomes considerably larger than that in the samples before annealing. This result corroborates the structural reconstruction from a partial segregation of Ge vacancies to a complete Ge gap in the Sb_2_Te_3_(GeTe)_17_ materials after the annealing. Furthermore, such a strong strain field would scatter the phonons substantially. According to the published literatures,^35^, ^36^ different scatters are effective of scattering phonons of different wavelength. For example, the long wavelength phonons are effectively scattered by the grain boundary scattering, while the short‐wavelength phonons are sensitive to the point defect scattering. Ge vacancies prefer to form the gap defects after long time annealing, and the reconstruction of the defect types gives rise to an extra effect on the phonon‐scattering process. Within Callaway model,^36^ we can write the associated relaxation time for the planar Ge vacancies as τ^−1^~*vσ*V_0_ too, where τ is the relax time and *v* is the sound velocity. And, in our case, σ = l*t where *l* is the characteristic length of the seen “linear” vacancies, *t* is the characteristic length of the “invisible” direction, *V*_0_ is the number density of this sort of planar vacancies = 1/l*t*d, where *d* is the characteristic distance between two nearest planar vacancies neighbors, as shown in the Figure S11 in the Supporting Information. We can thus rewrite the relaxation time to be τ^−1^~*v*/d. The characteristic distance between our two nearest planar vacancies *d* is about dozens to several hundred nanometers according to the histograms shown in Figure 3e–f. Although it might be weaker than grain boundaries, the scattering abilities of Ge vacancy gaps should not be neglected. Furthermore, with a scale of dozens to several hundred nanometers, the gaps scatter the long‐wavelength phonons effectively. GPA analysis also implied that the larger planar vacancies owns stronger strain field than the smaller planar vacancies, upon annealing. We thus think these vacancies with stronger strain field can scatter phonon much more effectively than those with weaker strain fields. Put it in another way, relaxation time due to planar vacancy scattering τ^−1^ = A*v*/*d*, although *d* is larger in annealed samples, but a larger scattering parameter *A* can make the compensation and eventually results in a smaller relaxation time τ thus stronger scattering efficacy of longer Ge gaps after annealing than short Ge gaps before annealing. This explains the observed lower lattice thermal conductivity after 7 days' annealing, as shown in Figure S5c in the Supporting Information. Note that we have expelled any ambiguousness originating from the possible grain growth during annealing processes. The fracture SEM images provided in Figure S12 in the Supporting Information exhibits that no apparently grain size growth after annealing. Besides the contrast of Ge gaps, there are also certain inclined striped‐like contrasts in Figure 3c,d, which is due to the intrinsic scanning distortions during the acquisition of STEM images. Such kind of contrast can be easily separated from the actual contrast due to strain as it is parallel to the scanning direction of electron beams, while the contrast corresponding to strain is strictly related to the Ge gaps. As a result, this artificial striped‐like contrast would not affect the conclusion from our GPA analysis.

The dynamics of Ge vacancies as a function of temperature are also explored by the in situ TEM and the results are given in Figure S9 in the Supporting Information. In this image, the STEM HAADF mode with a spatial resolution of ≈0.07 nm was used to obtain the more accurate details about the length and width of the planar defects. At room temperature, the defects are in a structure of short parallel planes consist of Ge vacancies. As temperature increases, these Ge vacancy planes tend to become more active and a huge growth of defects occurred at about 573 K, that is because it is approaching the phase transition temperature and the spontaneous adjustment process of the crystal structure gives a considerable space to the vacancies to move freely to a steady state. **Figure** **4**a–d shows the TEM diffraction contrast images of the sample at the high‐temperature region and it can be found directly that in this area there are lots of Ge vacancies at 673 K. However, when the temperature reached 773 K, large parts of them (marked by yellow arrows) disappear. When heating to 873 K, the concentration of Ge defects continues to decrease (marked by the blue arrows), just leaving several long‐range defects. These in situ TEM experiment results, which mimic the annealing process, are in consistent with the structure analysis on former part and reasonably explain that the annealing method is useful to optimize the densities and types of the Ge defects in this Sb_2_Te_3_(GeTe)_17_ system. As mentioned in the literatures,^16^ the high carrier concentration in the GST system is owing to the high Ge vacancy concentration. The dissociative cation defects prefer to recombine into the gaps during the annealing process makes a contribution on optimizing the carrier concentration. Then, the electrical property in this Sb_2_Te_3_(GeTe)_17_ system takes advantage of the decreasing carrier concentration during this vacancy engineering period.

**Figure 4:**
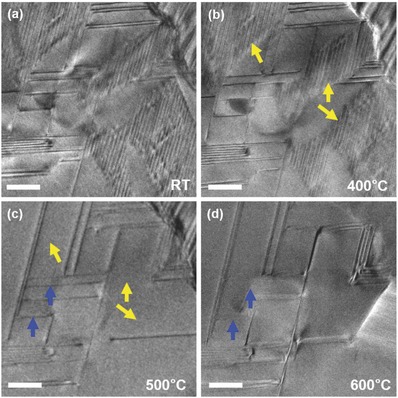
The in situ images in TEM model of a) no annealing Sb_2_Te_3_(GeTe)_17_ sample at room temperature, and b–d) the same area at 673, 773, and 873 K respectively. The scale bar is 50 nm.

Compared with reported GST samples,^30^, ^31^, ^32^ the performance of our annealed Sb_2_Te_3_(GeTe)_17_ samples is competitive (presented in **Figure** **5**a). A peak ZT value of 2.4 at 773 K has been achieved in our work, this value is the averaged value of three samples fabricated by the same process, and it is much higher than the reported value ≈2.0 for the Sb_2_Te_3_(GeTe)_17_ samples.^32^ It is said that the average ZT value (ZT_ave_) over the whole temperature range plays a more vital role than the peak ZT value in characterizing the conversion efficiency; therefore, the integrated area under the ZT curve can be used to calculate the ZT_ave_ value, and Figure 5b depicts the results. The ZT_ave_ of our Sb_2_Te_3_(GeTe)_17_ sample can reach 1.51 from 323 to 773 K, which is higher than previously work in the other Ge‐Sb‐Te based materials and with no doubt it is a record value. These statistical data show the huge applying potential of our annealed Sb_2_Te_3_(GeTe)_17_ thermoelectric materials.

**Figure 5:**
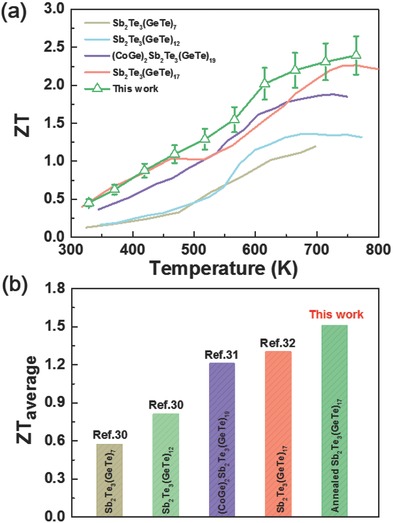
a) Comparison of ZT values of our work and reported samples in Ge‐Sb‐Te system. b) The average figure of merit ZT of our work and the reported samples.

In summary, pseudo‐layered Sb_2_Te_3_(GeTe)_17_ samples were proceeded with different annealing times, and the excellent high ZT value of 2.4 and ZT_ave_ of 1.51 was achieved with 7 days' annealing. We investigated the microstructural origin and found that intrinsic Ge vacancies in this Sb_2_Te_3_(GeTe)_17_ system migrated and reconstructed during the annealing progress, eventually forming a more stable defect type, i.e., the long range Van der Waals gaps. This microstructure reconstruction resulted in an abrupt decrease of carrier concentration, thus a sharp reduction of κ_e_. The steady high performance offered by Ge vacancies engineering provided a promising approach in the future research in GST materials.

## Experimental Section

*Sample Preparation*: A series of compounds Sb_2_Te_3_(GeTe)*_n_* (*n* = 7, 12, 14, 17, and 19) were mixed after stoichiometric weighing the elements of Ge, Sb, and Te. The samples were sealed in quartz tubes in a vacuum ≈10^−4^Pa and melted in the furnace. They were heated up to 673 K and kept for about 2 h, then slowly heated to 1223 K with 10 h and hold for another 2 h, followed by ice water quenching. The as‐obtained Sb_2_Te_3_(GeTe)_17_ ingots were then sealed in the tubes again to investigate the effect of annealing process, which were annealed at 873 K with 2, 4, and 7 days, respectively. The ramp‐up speed was 1 K min^−1^. These annealed ingots were hand milled to powder by the mortar and pestle after quenching in the ice water. The obtained powders were sintered at 823 K for 5 min under a uniaxial pressure of 50 MPa with a spark plasma sintering method (SPS‐211LX). To make sure the repeatability of the process, two more samples with the same method were made (annealing for 7 days).

*Physical Property Characterization*: The electrical conductivity (σ) and Seebeck coefficient (α) were simultaneously measured by the Ulvac Riko ZEM‐3 instrument with the uncertainty of ≈5% after cutting the samples into 2 × 2 × 12 cm^3^. The temperature‐dependent Hall coefficients (*R*_H_) were measured by a Hall Effect measurement system (Lake Shore 8400 Series), whose uncertainty was estimated to be 5%. The carrier concentration (*n*) was determined by 1/(*eR*_H_) and the carrier mobility (*µ*) was calculated as *σR*_H_. The thermal conductivity (κ) was calculated according to equation: *κ = DC*_p_ *d*. Here, *D* was the thermal diffusion coefficient and measured by a Netzsch LFA467 equipment from 300 to 773 K. *C*_p_ was the specific heat capacity estimated by Dulong–Petit law, which was equal to 0.237 J g^−1^K^−1^ for Sb_2_Te_3_(GeTe)_17_ composites. *d* was the density of each sample measured by the Archimedes' method. Considering the individual uncertainty of each parameter, the total uncertainty of the thermal conductivity was about 10%. All samples were characterized with a vacuum <10 Pa, which were limited by the instruments mentioned above.

*X‐Ray Diffraction and TEM*: The phases and structures of Sb_2_Te_3_(GeTe)_17_ were studied by XRD acquired with a Smartlab (9 kW, Rigaku) diffractometer equipment. A direct characterization of the samples' atomic structures and structural evolution during annealing process was carried out on a double *C*_S_‐corrected transmission electron microscopy FEI Titan G260‐300.

## Conflict of Interest

The authors declare no conflict of interest.

## Supporting information

Supplementary
