# Supplementary material for: Boosting the Thermoelectric Performance of Pseudo‐Layered Sb2Te3(GeTe)n via Vacancy Engineering
Source: Adv Sci (Weinh). 2018 Oct 12;5(12):1801514. doi: 10.1002/advs.201801514 (PMC6299710; doi:10.1002/advs.201801514)
Supplement: Supplementary file 1 — Supplementary [file ADVS-5-1801514-s001.pdf]

Copyright WILEY-VCH Verlag GmbH & Co. KGaA, 69469 Weinheim, Germany, 2018.

ADVANCED SCIENCE Open Access

Supporting Information

for *Adv. Sci.,* DOI: 10.1002/advs.201801514

Boosting the Thermoelectric Performance of Pseudo-Layered $Sb_2Te_3(GeTe)_n$ via Vacancy Engineering

*Xiao Xu, Lin Xie, Qing Lou, Di Wu, and Jiaqing He**

Supporting Information

Boosting the Thermoelectric Performance of Pseudo-layered $Sb_2Te_3(GeTe)_n$ *via* Vacancy Engineering

*Xiao Xu, Lin Xie, Qing Lou, Di Wu, and Jiaqing He**

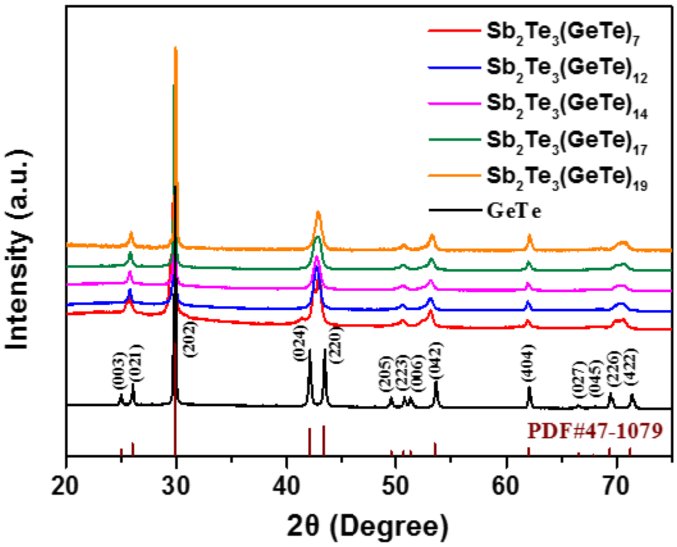

Figure S1. XRD patterns of $Sb_2Te_3(GeTe)_n$ samples.

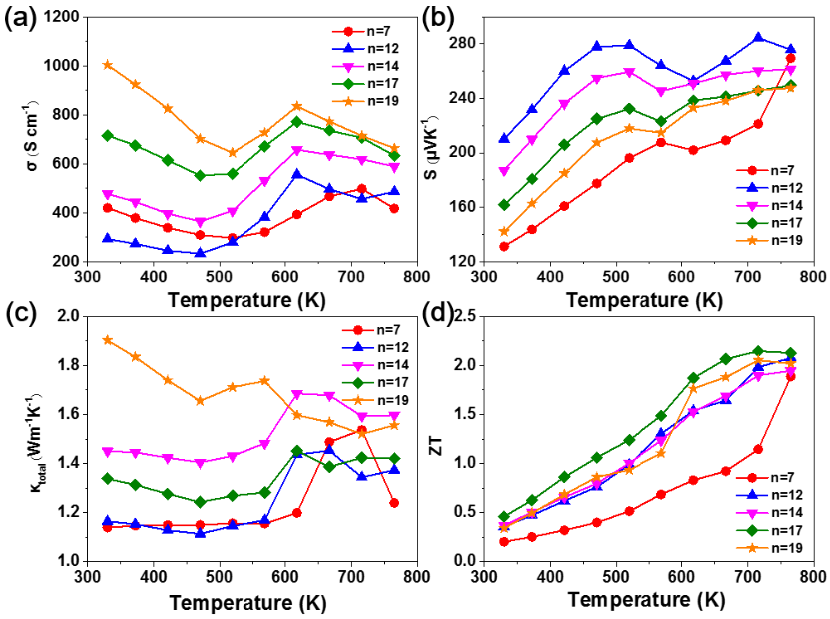

Figure S2. Thermoelectric performance of $Sb_2Te_3(GeTe)_n$ samples, (a) electrical conductivity, (b) Seebeck coefficient, (c) total thermal conductivity and (d) *ZT* value.

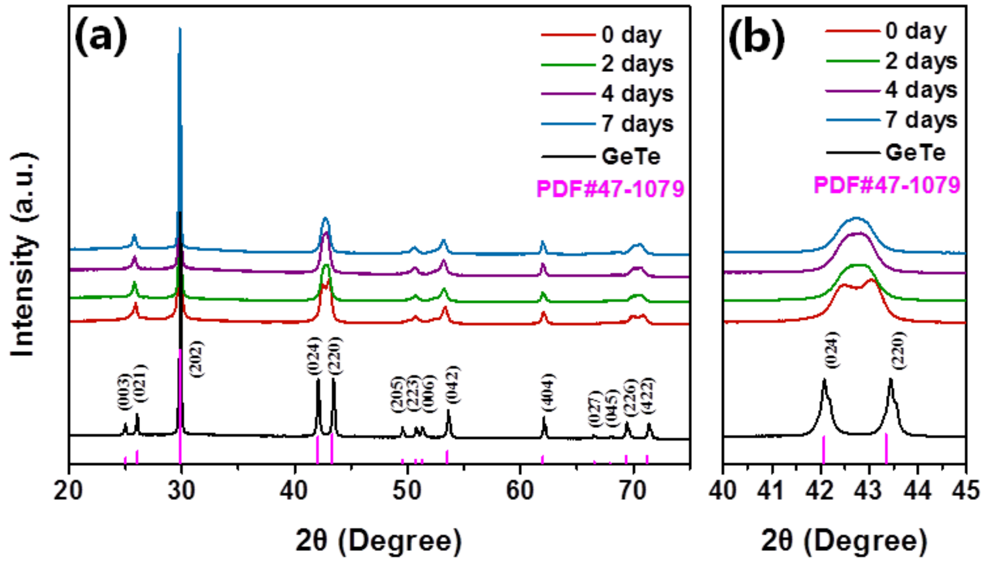

Figure S3. (a) XRD pattern and (b) the magnified XRD pattern of the $Sb_2Te_3(GeTe)_{17}$ samples (annealing for 0~7 days).

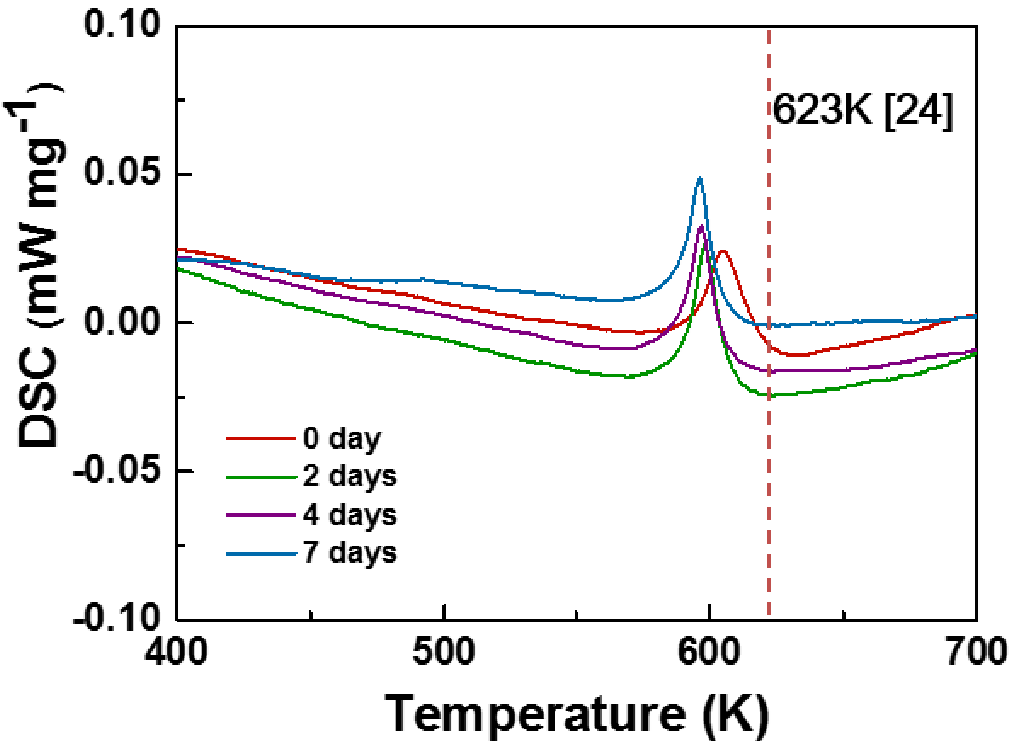

Figure S4. The temperature dependence of differential scanning calorimetry curves of the four $Sb_2Te_3(GeTe)_{17}$ samples.

Analysis for Figures S3 and S4: The XRD patterns of our $Sb_2Te_3(GeTe)_{17}$ samples are illustrated in Figure S3 and the data are normalized for comparison. All the samples show the same single phase without impurities. Comparing our XRD results with GeTe which fabricated by the same method, it seems like that some peaks of GeTe tend to merge into one peak, suggesting our GST materials have a higher symmetry than that of the rhombohedral phase as expected for GeTe-based materials at room temperature. Especially from 41 degree to 44 degree (as is magnified in Figure S3 (b)), the merging of peaks becomes more evident as the annealing time increases. Generally speaking, structure with higher symmetry has better electronic transport properties, suggesting that increasing the annealing time can enhance the electrical performance. To highlight the advantages of this structure, the differential scanning calorimetry (DSC) curves have been given in Figure S4. The phase transition temperature of $Sb_2Te_3(GeTe)_{17}$ samples are much lower than those of pure GeTe (T_c~700K) and the tuned $Ge_{0.87}Pb_{0.13}Te$+3%Bi_2Te_3 (T_c~623K) sample[24], indicating that the low temperature phase of $Sb_2Te_3(GeTe)_{17}$ system requires less energy to transfer into high temperature cubic phase, and this is of great benefit to the enhancement of properties according to the literature[33]. Furthermore, the phase transition temperature of the annealing samples decreases with increasing annealing time, suggesting that annealing method is effective for the reduction of T_c, which is consistent with the XRD results.

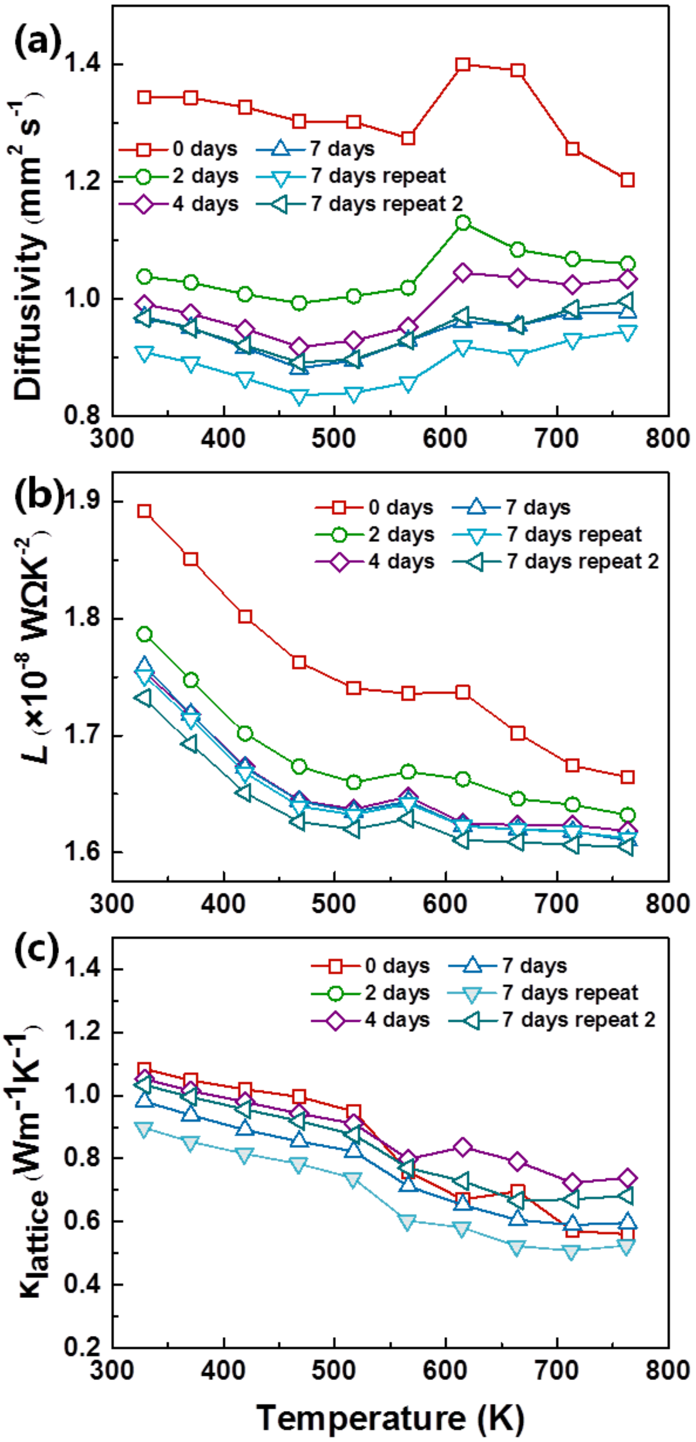

Figure S5. The temperature dependence of (a) diffusivity, (b) Lorenz number and (c) lattice thermal conductivity of the $Sb_2Te_3(GeTe)_{17}$ samples (annealing for 0~7 days) and the refabricated samples.

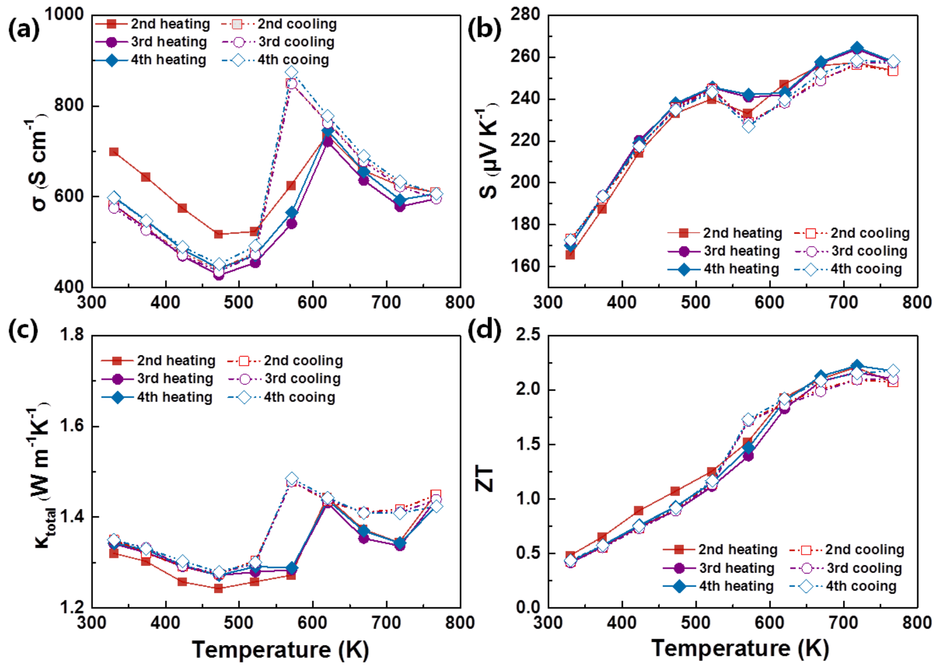

Figure S6. The temperature dependence of (a)the electrical conductivity, (b) the Seebeck coefficient, (c) the power factor and (d) the total thermal conductivity of the 7 days' annealing $Sb_2Te_3(GeTe)_{17}$ sample with three times heating and cooling.

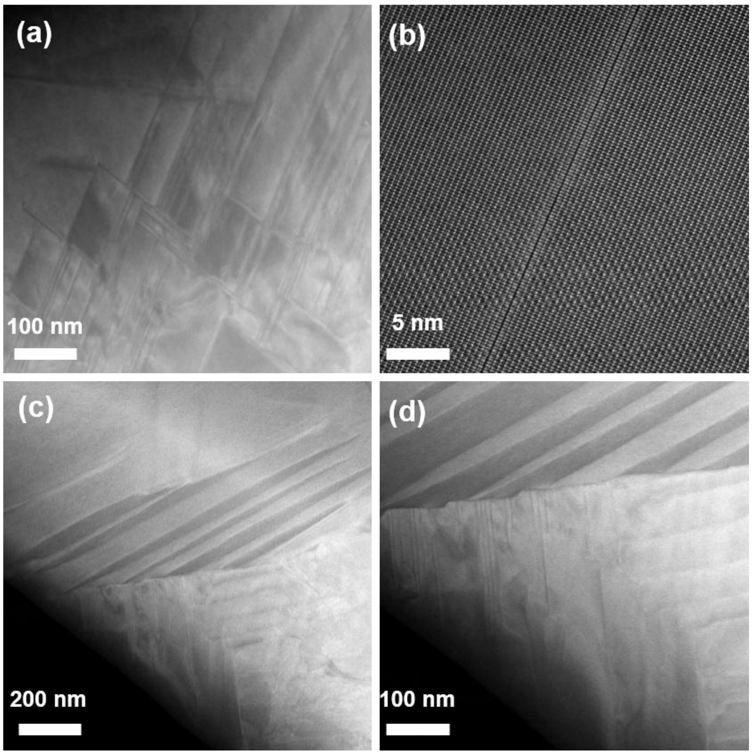

Figure S7. The TEM images of the annealed $Sb_2Te_3(GeTe)_{17}$ sample. (a) the low magnified images from another direction, (b) the high magnified images from the same direction with (a), (c) and (d) are the low magnified images of two crystal grains.

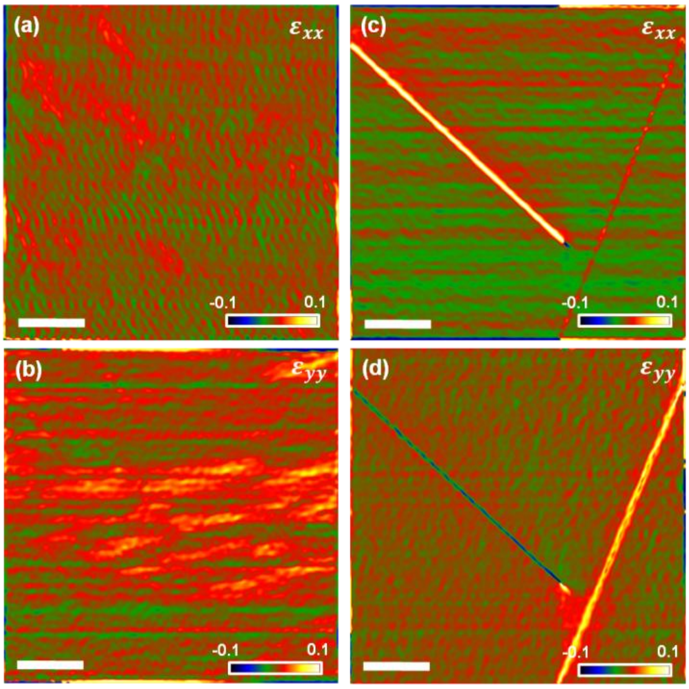

Figure S8. The GPA strain analysis images of the same region of the HRTEM images in the no annealing samples in (a) e_{xx} and (b) e_{yy} and 7 days' annealing sample in (c) e_{xx} and (d) $e_{yy.}$ The scale bar was 10 nm.

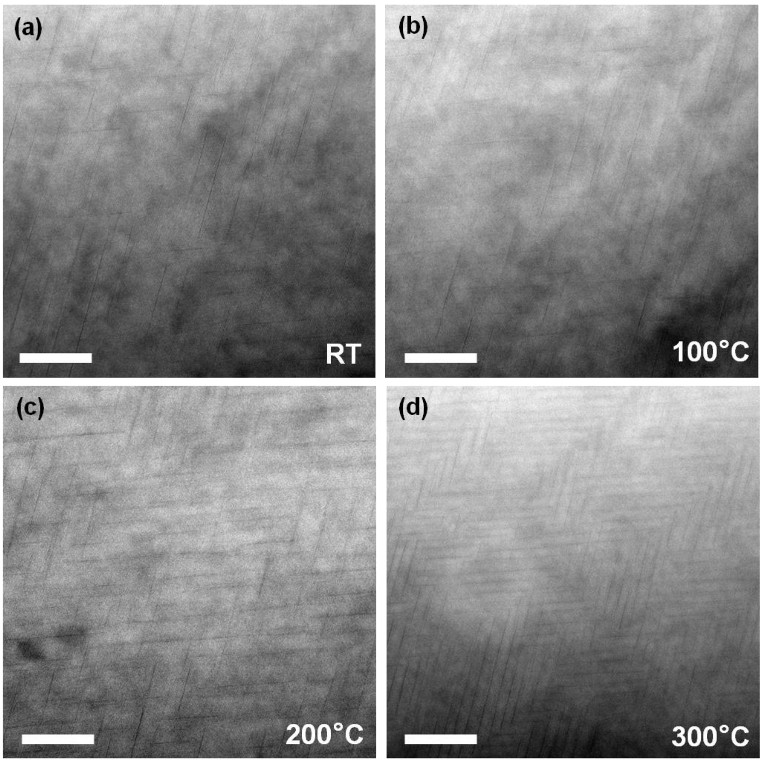

Figure S9. The *In situ* TEM images of the no annealing $Sb_2Te_3(GeTe)_{17}$ sample from room temperature to 573 K. The scale bar was 20 nm.

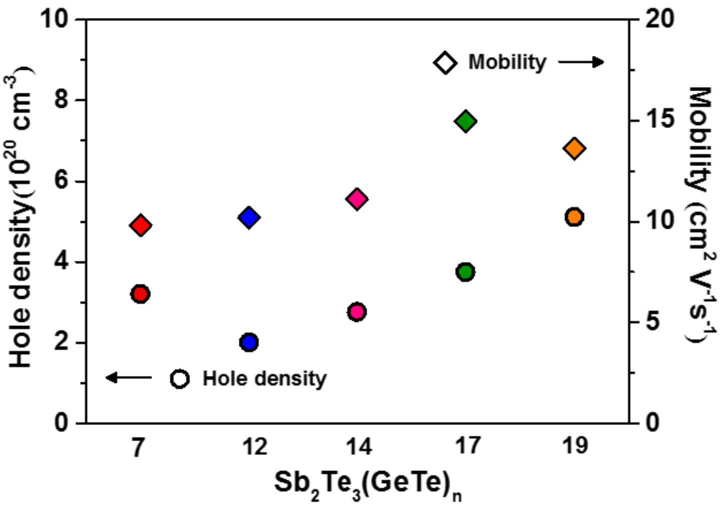

Figure S10. The n dependence of hole density and mobility for $Sb_2Te_3(GeTe)_n$ samples at room temperature.

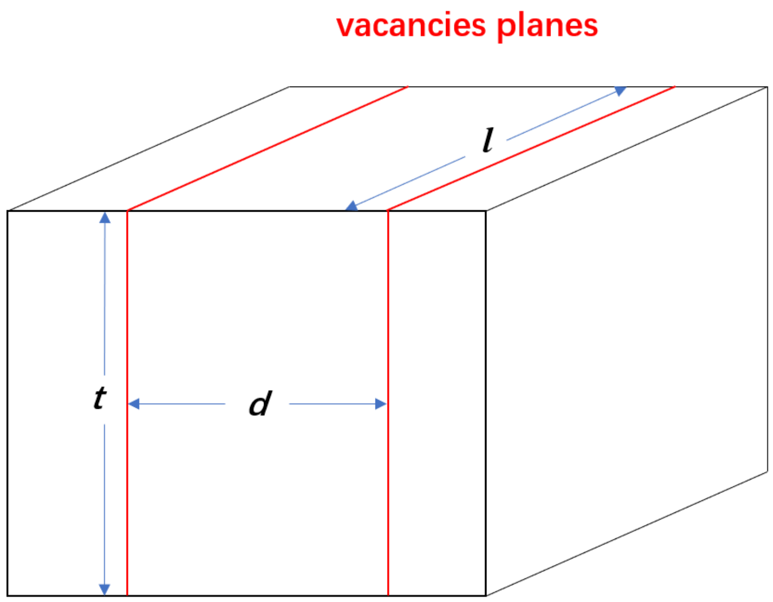

Figure S11. The sketch of the characteristic distance d between two planar vacancies. Here, *l* is the characteristic length of the seen "linear" vacancies, t is the characteristic length of the "invisible" direction.

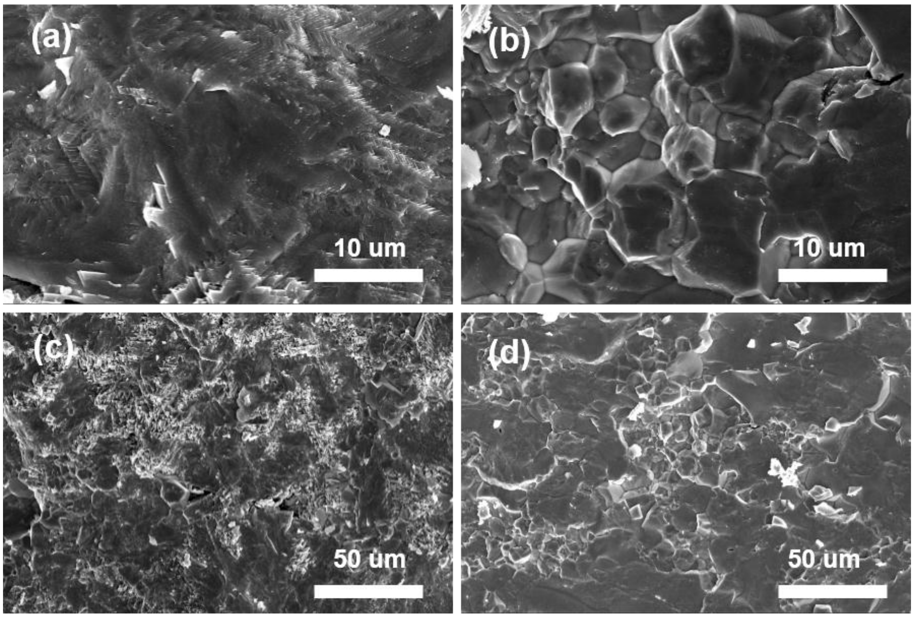

Figure S12. The fracture SEM images of the (a) non-annealing $Sb_2Te_3(GeTe)_{17}$ sample. (b) 7 days' annealing $Sb_2Te_3(GeTe)_{17}$ sample. Lower magnified SEM images of the (c) non-annealing $Sb_2Te_3(GeTe)_{17}$ sample, and (d) 7 days' annealing $Sb_2Te_3(GeTe)_{17}$ sample.

Anisotropy

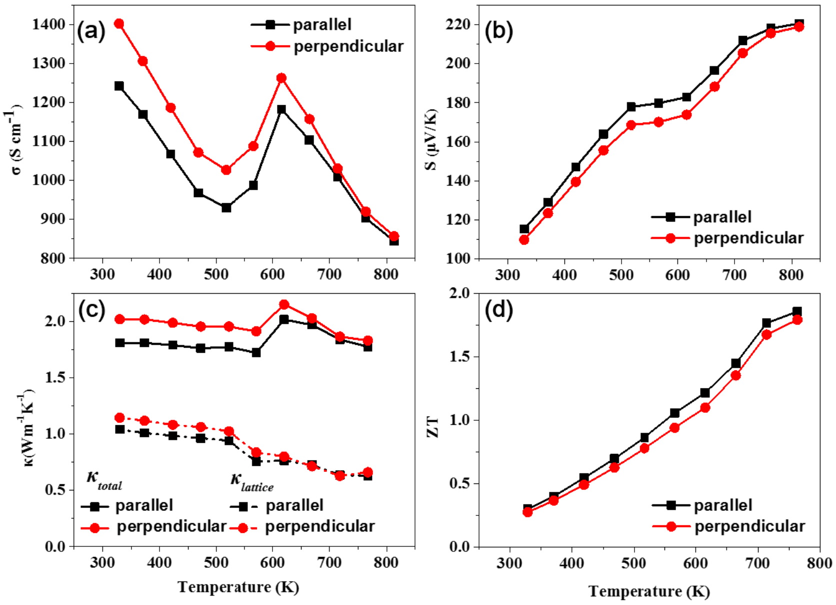

Figure S13. The Thermoelectric performance along the parallel and perpendicular direction in $Sb_2Te_3(GeTe)_{17}$ samples, (a) electrical conductivity, (b) Seebeck coefficient, (c) thermal conductivity and (d) *ZT* value.
